# DETECTING COGNITIVE IMPAIRMENT FROM DIGITAL VOICE RECORDINGS OF A REPETITION TASK

**DOI:** 10.1093/geroni/igad104.0046

**Published:** 2023-12-21

**Authors:** Stacy Andersen, Nicole Roth, Seho Park, Thomas Perls, Stephanie Cosentino, Rhoda Au, Paola Sebastiani

**Affiliations:** Boston University Chobanian & Avedisian School of Medicine, Boston, Massachusetts, United States; Boston University School of Medicine, Boston, Massachusetts, United States; Boston University Chobanian & Avedisian School of Medicine, Boston, Massachusetts, United States; Boston University, Boston, Massachusetts, United States; Columbia University Medical Center, New York City, New York, United States; Boston University Chobian & Avedisian School of Medicine, Boston, Massachusetts, United States; Tufts Medical Center, Boston, Massachusetts, United States

## Abstract

Early detection of Alzheimer’s disease is needed but cognitive screeners, used widely in research and clinical settings, suffer from poor sensitivity and specificity. Acoustic features of speech are altered in Alzheimer’s disease and may aid detection of cognitive impairment. We sought to test whether acoustic metrics from a cognitive screener differentiate individuals with cognitive impairment from those who are cognitively normal. We extracted 38 acoustic features from digital voice recordings of the Mini-Mental Status Examination repetition task from Long Life Family Study participants using the openSMILE toolkit. Highly correlated features (r>0.9) were removed. Cognitive status (impaired versus cognitively normal) was determined through consensus review of neuropsychological testing and informant interviews. Bayesian Information Criterion-based stepwise regression including 24 acoustic variables and retaining age, sex, education, and MMSE score in all models was used to identify predictors of cognitive status. Acoustic features from preliminary data included 277 participants (mean age 74.8±12.3 years old, 57% females, 34% cognitively impaired due to oversampling). Stepwise selection identified greater loudness (i.e., signal intensity; p<0.001), smaller pitch changes (p=0.007), and lower Harmonics-to-Noise Ratio (i.e., a metric of voice quality; p=0.017) as significantly associated with cognitive impairment. A model with MMSE, demographics, and acoustics had a higher discriminative value for cognitive status (AUC 0.95, sensitivity=0.97, specificity=0.73) than the model without acoustics (AUC = 0.94, sensitivity=0.97, specificity=0.63). Overall, acoustic features from a brief (i.e., 3 second) repetition task were associated with cognitive impairment and added information beyond traditional scoring that increased the specificity of a cognitive screener.

